# Improved Insulin Resistance and Lipid Metabolism by Cinnamon Extract through Activation of Peroxisome Proliferator-Activated Receptors

**DOI:** 10.1155/2008/581348

**Published:** 2008-12-11

**Authors:** Xiaoyan Sheng, Yuebo Zhang, Zhenwei Gong, Cheng Huang, Ying Qin Zang

**Affiliations:** Key Laboratory of Nutrition and Metabolism, Institute for Nutritional Sciences, Shanghai Institutes for Biological Sciences, Graduate School of CAS, Chinese Academy of Sciences, 319 Yue Yang Road, Shanghai 200031, China

## Abstract

Peroxisome proliferator-activated receptors (PPARs) are transcriptional factors involved in the regulation of insulin resistance and adipogenesis. Cinnamon, a widely used spice in food preparation and traditional antidiabetic remedy, is found to activate PPAR*γ* and *α*, resulting in improved insulin resistance, reduced fasted glucose, FFA, LDL-c, and AST levels in high-caloric diet-induced obesity (DIO) and *db*/*db* mice in its water extract form. In vitro studies demonstrate that cinnamon increases the expression of peroxisome proliferator-activated receptors *γ* and *α* (PPAR*γ*/*α*) and their target genes such as LPL, CD36, GLUT4, and ACO in 3T3-L1 adipocyte. The transactivities of both full length and ligand-binding domain (LBD) of PPAR*γ* and PPAR*α* are activated by cinnamon as evidenced by reporter gene assays. These data suggest that cinnamon in its water extract form can act as a dual activator of PPAR*γ* and *α*, and may be an alternative to PPAR*γ* activator in managing obesity-related diabetes and hyperlipidemia.

## 1. INTRODUCTION

Obesity has become the most common metabolic disorder in the world and is a major risk factor for insulin resistance in the development of type 2 diabetes mellitus [1]. Peroxisome proliferator-activated receptors (PPARs) have been recognized as therapeutic targets against dyslipidemia and diabetes since their discovery in the early 1990s. PPARs are ligand-activated nuclear hormone receptors that include three isoforms: PPAR*α*, PPAR*γ*, and PPAR*δ*/*β*. PPAR*α* is expressed mostly in brown adipose tissue and liver, PPAR*γ* is mainly expressed in adipose tissue, while PPAR*δ*/*β* is expressed universally in many tissues [2]. Activation of PPAR*α* lowers plasma triglycerides and elevates plasma HDL cholesterol levels [3], while the activation of PPAR*γ* increases insulin sensitivity and results in antidiabetic effects [4]. Since PPARs play key roles in regulating carbohydrate and lipid metabolism, most studies have been directed toward developing synthetic PPAR ligands, and current therapeutic strategies are also based on separate treatments of insulin resistance and dyslipidaemia. Fibrates, the PPAR*α* agonists, are now recommended for the treatment of cardiovascular disease and dyslipidemia [5]. Thiazolinediones (TZDs), the PPAR*γ* agonists, have been demonstrated by a number of clinical trials in the management of insulin resistance and type 2 diabetes [6–8]. However, these agonists can also produce moderate to serious side effects such as edema, weight gain, congestive heart failure, and hepatotoxicity [9, 10]. Thus, the development of drugs of dual or pan PPAR modulators with less side effects is desirable for the management of obesity-related diabetes and dyslipidemia.

Cinnamon is the bark of *Cinnamoni cassiae* and has been used as traditional folk herbs to treat inflammation for thousands of years in Asia. It is also used in food industry as antioxidant and spicy agent. In recent years, several studies have reported that cinnamon extract has antidiabetic effect on *db*/*db* mice and type 2 diabetic patients [11, 12]. Talpur et al. showed that cinnamon oils can improve insulin sensitivity [13] and Roffey et al. reported that cinnamon water extract (CE) increases glucose uptake in 3T3-L1 adipocyte [14]. However, the separated compounds derived from cinnamon displayed little insulin-like or insulin-enhancing activity [15]. In this report, we demonstrate that cinnamon water extract can elevate the expression of both PPAR*γ*, *α* and their target genes in 3T3-L1 adipocyte possibly through stimulation of the transactivities of both full length and LBD of PPAR*γ* and PPAR*α*. In vivo study reveals that cinnamon water extract improves insulin resistance and lipid metabolism in both high-calorie diet-induced obesity (DIO) mice and *db*/*db* mice.

## 2. MATERIALS AND METHODS

### 2.1. Materials

Dexamethasone (DEX), 3-isobutyl-1-methylxanthine, insulin, oil red O, troglitazone, and WY-14643 were purchased from Sigma (St. Louis, Mo). Cinnamon powder was obtained from Shanghai Traditional Chinese Herbs Pharmacy Co. (Shanghai, China). The power (10 g) was extracted in (100 mL) double distilled water with revolving evaporator in vacuum state using vacuum pump till the volume of water reduced to 50 mL. The supernatant was then filtered through Whatman paper no. 1 to obtain the cinnamon water extract. The final concentration was 0.2 g/mL.

### 2.2. Quantitative RT-PCR

Total RNA extracted from 3T3-L1 adipocyte or mouse tissues was reverse transcribed into first strand cDNA with random hexamer primers using a cDNA synthesis kit (Promega, Madison, Wis). The gene expression levels were analyzed by quantitative real-time RT-PCR conducted with the ABI 7500 (Applied Biosystems, Foster City, Calif). After an initial incubation for 2 minutes at 50°C, the cDNA was denatured at 95°C for 10 minutes followed by 40 cycles of PCR (95°C, 15 seconds, 60°C, 60 seconds). All results were obtained in at least three independent experiments. The mRNA levels of all genes were normalized using *β*-actin as an internal control.

### 2.3. Luciferase activity assays

The plasmid construction and transfection were carried out as described previously [16]. Briefly, 293T cells obtained from ATCC were cultured in DMEM with 10% FBS. Transfection solution was added to 293T cells overnight, then CE and PPAR ligands were added into fresh media and the cells were harvested for the determination of luciferase activity 24 hours later. The luciferase reporter assays were carried out using the Dual-Luciferase Reporter Assay System (Promega, Madison, Wis) and the transfection efficiencies were normalized by renilla luciferase activity. All transfection experiments were performed in duplicate and repeated at least three times independently.

### 2.4. 3T3-L1 adipocyte differentiation and oil red O staining

3T3-L1 cells were purchased from American Type Culture Collection (Va, USA) and maintained in DMEM containing 10% FBS. To induce differentiation, two days after confluence, preadipocytes (designated day 0) were cultured in differentiation medium (DM, containing 10% FBS plus 1 *μ*g/mL insulin, 0.5 *μ*M dexamethasone, 0.5 mM IBMX) or DM + CE at indicated concentrations for 2 days, then switched to post DM containing 10% FBS and 1 *μ*g/mL insulin. The medium was changed every 2 days. Oil red O staining was performed on day 5. Briefly, cells were washed twice with PBS and fixed with 7.5% formaldehyde for 15 minutes. After twice washing with PBS, cells were stained for at least 1 hour in freshly diluted oil red O solution in 37°C. Cells were photographed using a phase-contrast Olympus IX70 (Tokyo, Japan) microscope in combination with a MicroFire digital camera at 200 magnifications.

### 2.5. Western blot analysis

Cells were homogenized in 2X SDS sample buffer (Invitrogen, Carlsbad, Calif) and boiled for 5 minutes. 10 *μ*g of protein was separated by 10% SDS-PAGE and transferred to PVDF membrane, blocked for 1 hour at room temperature in 5% nonfat dry milk/PBS-T. PPAR*γ* (Upstate, Charlottesville, Va) and *β*-actin antibodies (Sigma, St. Louis, Mo) were added to 2% BSA in PBS-T and incubated with the membrane for 1 hour at room temperature. The membrane was then washed 3 times with PBS-T for 10 minutes each, incubated with HRP conjugated secondary antibody (Santa Cruz, Calif) for 30 minutes at room temperature, and washed 3 times with PBS-T for 30 minutes totally. The signals were detected by ECL Plus Western Blotting Detection System according to the manufacturer’s specifications (Amersham, Little Chalfont Buckinghamshire, England).

### 2.6. In vivo animal study

Female C57BL/6J mice were purchased from the SLAC Laboratory (Shanghai, China) and *db*/*db* (C57BL BKS cg-M^+/+^ lepr^−/−^) mice were purchased from Jackson Lab (Me, USA) and bred in house. All mice were kept under controlled temperature (22-23°C) and on a 12-hour light/dark cycle. C57BL/6J mice were placed on a high-fat diet (60% of calories derived from fat, Research Diets, New Brunswick, NJ, D12492) for induction of obesity, while control mice were placed on the equivalent chow diet (10% of calories derived from fat, Research Diets, D12450B). DIO mice with similar body weight and *db*/*db* mice with similar fasted glucose level were used in the experiments. Mice were gavaged with CE (correspond to 400 mg cinnamon powder kg • day •) or water only as control at 2PM each day in a vehicle of water. All animal protocols used in this study were preapproved by Shanghai Institutes for Biological Sciences of CAS for animal studies.

### 2.7. Intraperitoneal glucose tolerance test (IPGTT)

C57BL/6J mice were fasted overnight on day 21 and *db*/*db* mice were fasted for 6 hours on day 15 prior to the blood samples were collected from tail vein for the determination of baseline glucose values (*t* = 0 minute) before the injection of glucose (intraperitoneal injection 2 g/kg). Additional blood samples were collected with regular intervals (*t* = 15, 30, 60, 90, 120, and 240 minutes) for glucose measurement. Blood glucose levels were evaluated with a glucose analyzer (TheraSense, Abbott Park, Ill).

### 2.8. Serum chemistry analysis

Serum triglyceride (TG), total cholesterol (TC), HDL-C and LDL-C, AST, and ALT were examined using a Hitachi 7020 Automatic Analyzer in 100 *μ*l of heart blood serum. FFA was analyzed with an Ultrospec 2100 Pro UV/Visible spectrophotometer (Amersham, Little Chalfont Buckinghamshire, England) with kits from Jiancheng Bioengineering Ins (Nanjing, China). Serum insulin was measured by ELISA kit (Linco Research, Mo, USA).

### 2.9. Statistical analysis

The results were represented as the mean ± SE. Data were analyzed by the statistical analysis system (SAS) program. Comparison between the control and treated groups was made by ANOVA variance analysis and their significance was established by Student’s *t*-test. Differences of *P* < .05 were considered statistically significant.

## 3. RESULTS

### 3.1. CE improves hyperlipidemia and liver function in DIO C57BL/6J and *db/db* mice

After 5-month induction with high-fat diet, the body weight of C57BL/6J mice was increased to 150% and the serum TG, LDL, AST, and ALT were greatly elevated compared to normal food fed mice as indicated in Table 1. Three weeks after gavage treatment with CE, equivalent to cinnamon 400 mg/kg • day, serum FFA and LDL-c were significantly reduced (see Table 1). However, the body weight and food intake remained unchanged (data not shown). Since high-fat diet can usually impair the liver function and elevate AST and ALT levels, we examined AST and ALT in CE-treated DIO mice and found that CE intervention can effectively improve liver function as evidenced by significantly reduced AST and ALT levels in DIO mice (see Table 1). To testify whether CE has similar effects on *db*/*db* mice, 11 weeks old *db*/*db* mice were treated with CE for 2 weeks. The serum FFA of *db*/*db* mice was decreased markedly after 2 weeks of CE treatment (data not shown), suggesting that CE can also improve dyslipidemia in genetically defective obese mice.

### 3.2. CE reduces hyperglycemia and improves glucose tolerance in both DIO mice and *db/db* Mice

**Figure 1:**
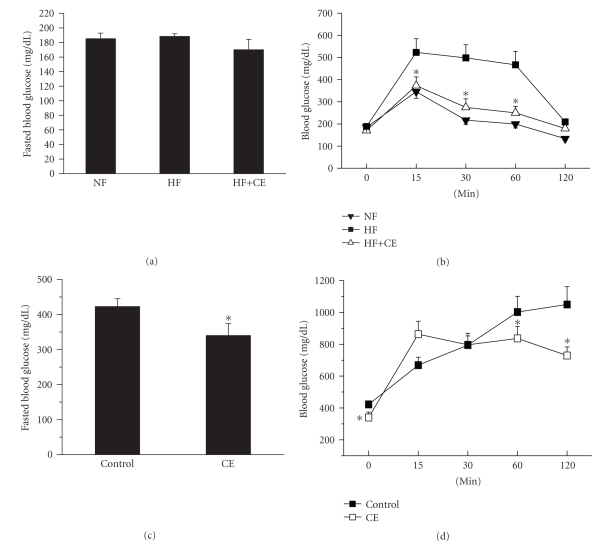
CE reduced the fasted blood glucose level and improved glucose tolerance in DIO and *db*/*db* mice. Mice were administered vehicle alone, or CE (equivalent to 400 mg cinnamon powder kg • day •) for 3 weeks (DIO) or 2 weeks (*db*/*db*). The blood glucose levels were measured in tail vein and the basal glucose levels were shown at 0 minute. (a) DIO C57BL/6J mice were fasted overnight and glucose levels were measured at 9:00 am at the end treatment. NF: normal food fed mice; HF: high-fat food-induced mice; CE: cinnamon water extract; (b) fasted DIO mice were intraperitoneally injected with 2 g glucose • kg^−^ body weight and glucose tolerance determined at the time indicated; (c) *db*/*db* Mice were fasted for 6 hours and the fasted blood glucose was measured. CONT: vehicle; (d) glucose tolerance was determined following 2 g glucose • kg^−1^ body weight intraperitoneal injection in *db*/*db* mice. The data are presented as mean ± SE, *n* = 5 for each group. (b) *P < .05 versus HF group or (d) vehicle control (cont).

To evaluate whether CE has any influence on glucose tolerance, IPGTT determination was carried out for overnight fasted DIO mice at 0 minute, 15 minutes, 30 minutes, 60 minutes, and 120 minutes following 2 g/kg glucose intraperitoneal injection DIO mice developed severe insulin resistance after 5 months high-fat food inductions, and serum insulin level increased nearly 3 times compared to the control mice on normal diet (see Table 1). Three weeks of CE treatment not only reduced fasted glucose level but also improved glucose tolerance markedly (see Figures 1(a) and 1(b)). The serum insulin level was decreased after CE treatment (Table 1). Similar effects were observed on *db*/*db* mice as demonstrated in Figures 1(c) and 1(d). Our results suggested that CE is capable of improving insulin sensitivities in both high-fat diet-induced diabetic mice and *db*/*db* mice.

### 3.3. CE promotes 3T3-L1 preadipocyte differentiation

**Figure 2:**
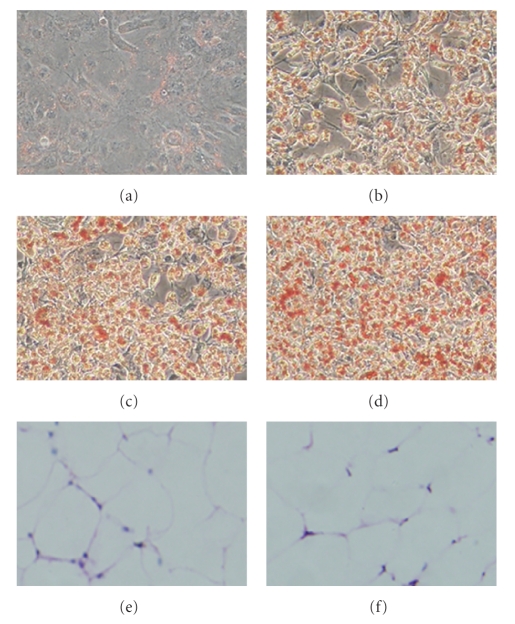
CE promoted 3T3-L1 adipocyte differentiation. 3T3-L1 cells were stained with oil red O at day 5. (a) Undifferentiated control cells; (b) DM (insulin 10 *μ*g/mL, dexam: 1 *μ*M, IBMX: 0.5 mM)-induced differentiated cells; (c) DM-induced differentiated cells + CE 0.2 mg/mL; (d) DM-induced differentiated cells + CE 0.6 mg/mL; (e) HE staining of WAT from high-fat diet control (HFC) mice; (f) HE staining of WAT from HF + CE-treated mice.

In order to understand the underlying mechanism of CE in hypolipidemia and hypoglycemia, we used preadipocyte 3T3-L1 as an in vitro system to study the influence of CE on preadipocyte differentiation. To analyze the effects of CE on 3T3-L1 preadipocyte differentiation, CE was added to 3T3-L1 cells during differentiation induction in differentiation medium (DM). On day 5, we found that the number and the volume of adipocytes were increased in CE-treated cells compared to those induced by DM only indicating that CE may promote 3T3-L1 preadipocyte differentiation (see Figures 2(a), 2(b), 2(c), and 2(d)). However, the number and size of the adipocytes in WAT in CE-treated DIO mice remain unchanged (Figures 2(e) and 2(f)).

### 3.4. CE enhances gene expression of PPAR*γ*, PPAR*α*, and the target genes

**Figure 3:**
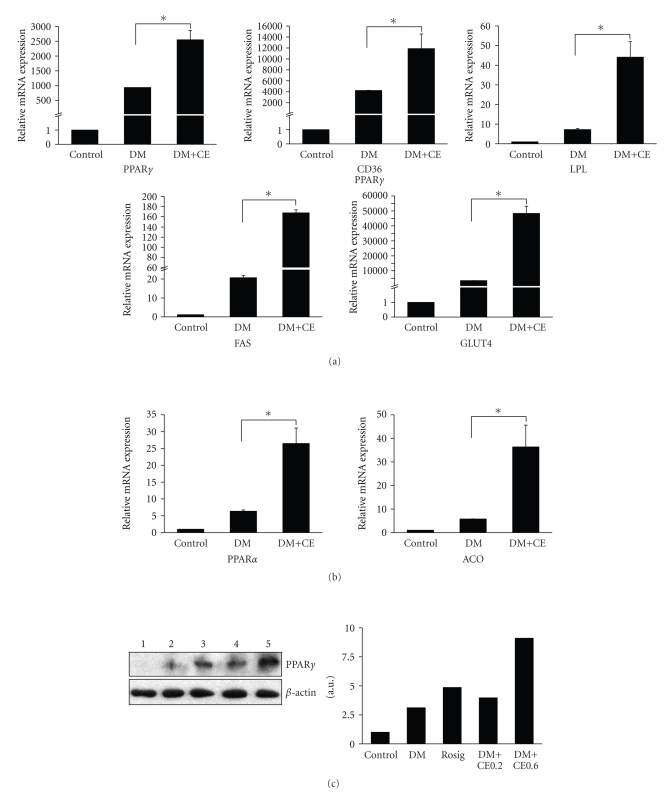
CE increased expression of PPARs and their target genes in differentiated 3T3-L1 cells. 3T3-L1 cells were differentiated in 24-well plate and CE 0.6 mg/mL was added at the same time. On day 5, cells were collected and total RNA was extracted and reversely transcribed into the first strand cDNA with random hexamer primers using cDNA synthesis kit. The gene expression levels were analyzed by quantitative real-time RT-PCR. (a) PPAR*γ* and its target genes; (b) PPAR*α* and its target gene; (c) Western blot of CE-treated 3T3-L1 differentiated cells from day 5. 1: Control; 2: DM; 3: Rosiglitazone 1 *μ*M; 4: DM + CE 0.2 mg/mL; 5: DM + CE 0.6 mg/mL. For real-time PCR, the results were repeated in at least 3 independent experiments, and *β*-actin mRNA was used as an internal control. Data are presented as mean ± SE. *P < .001.

Since PPAR*γ* and PPAR*α* play essential roles in adipocyte differentiation and lipid homeostasis, we further studied whether CE increases 3T3-L1 adipocyte differentiation through the activation of PPARs. The above-mentioned CE-treated cells were collected to determine the expression levels of PPAR*γ*, PPAR*α* and their target genes such as CD36, FAS, LPL, GLUT4, and ACO. The results showed that CE not only elevated the expression of PPAR*γ* and its target genes CD36, LPL, FAS, and GLUT4 significantly (see Figure 3(a)), but also increased the expression of PPAR*α* and its target gene ACO markedly (Figure 3(b)). Further examination at the protein level of PPAR*γ* confirmed that CE can significantly increase the PPAR*γ* protein level in 3T3-L1 adipocyte during differentiation (Figure 3(c)).

### 3.5. CE increases transactivities of PPAR*γ* and PPAR*α*

**Figure 4:**
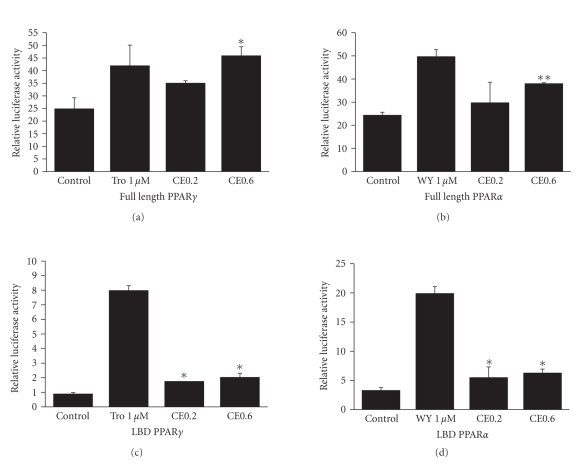
CE activated transactivities of PPAR*γ* and PPAR*α*. Full-length PPAR*γ* or PPAR*α* was cotransfected with PPRE-J3-TK-Luc reporter construct to 293T cell and treated with CE (0.2 mg/mL, 0.6 mg/mL), 1 *μ*M of troglitazone or WY-14643 for 24 hours as positive controls. Rellina luc was used as a transfection efficiency control and the relative luciferase activities were measured against renilla luciferase activities. For LBD activity assay, pMCX-GAL4-LBD of PPAR*γ* or *α* expression constructs were cotransfected with USA_G_ × 4-TK-Luc into 293T using the same protocol as described above. The empty vectors were used as control. (a) Full-length PPAR*γ*. (b) Full-length PPAR*α*. (c) LBD PPAR*γ*. (d) LBD PPAR*α*. The results represent three independent experiments. Data are presented as mean ± SE. *P < .05, **P < .01. Tro: troglitazone; WY: WY14643.

To test whether CE could increase the transactivities of PPAR*γ* and PPAR*α*, full-length PPAR*γ* and PPAR*α* expression plasmids were transfected into 293T cells for the reporter gene assays. We found that CE increased the transactivities of both PPAR*γ* (*P* < .05) and PPAR*α* (*P* < .01) in a dose-dependent manner (see Figures 4(a) and 4(b)). In order to exclude the endogenous factors, ligand-binding domains (LBD) of PPAR*γ* and PPAR*α* were used to evaluate the reporter gene activities. Results showed that the LBD activities of both PPAR*γ* and PPAR*α* were also elevated (*P* < .05) (see Figures 4(c) and 4(d)). These data suggest that CE is a dual activator of both PPAR*γ* and PPAR*α*.

### 3.6. CE treatment elevates the expression of PPAR*γ*/*α* and target genes in DIO mice

**Figure 5:**
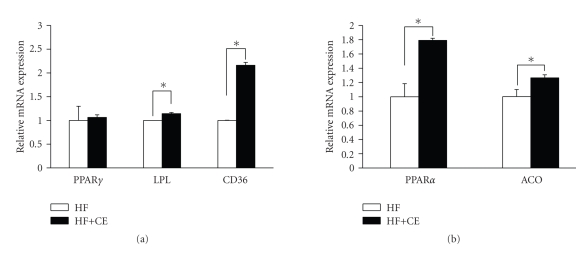
CE promoted gene expression of PPAR*γ*, PPAR*α* and their target genes in WAT and liver from DIO mice: (a) real-time PCR of PPAR*γ* and its target gene expression in white fat tissue; (b) real-time PCR of PPAR*α* and its target gene expression in liver. HF: high-fat diet; *n* = 5; data are presented as mean ± SE. *P < .05 compared to control.

To verify whether CE treatment has any effect at the transcriptional level in related tissues in DIO mice, we examined the gene expression of PPARs and their target genes in white adipocyte tissue (WAT) and liver of CE-treated DIO mice and controls. PPAR*γ*, dominantly expressed in WAT, was slightly elevated, but its target genes, LPL and CD36, were increased markedly (see Figure 5(a)). PPAR*α* and its target gene ACO, mainly expressed in liver, were also elevated significantly in CE-treated mice compared with the controls (Figure 5(b)). In vivo data further suggested that CE is a dual activator of PPAR*γ* and PPAR*α*.

## 4. DISCUSSION

There are several studies reported that cinnamon has anti-hyperglycemic and anti-hyperlipidemic effects on diabetic animals and type 2 diabetic patients. However, the underlying mechanisms remain illusive. Here, we use early type 2 diabetic DIO mice, and severe type 2 diabetic *db*/*db* mice, as in vivo models, and 3T3-L1 adipocyte as in vitro differentiation model to study the effects of CE on lipid and glucose metabolism and their possible underlying mechanisms.

In vitro studies demonstrate that CE acts like a dual activator to PPAR*γ*/*α* based on three evidences: first, oil red O staining showed that CE stimulated differentiation of 3T3-L1 cells from preadipocytes into adipocytes. Secondly, CE treatment not only increased mRNA levels of PPAR*γ* and PPAR*α* but their target genes CD36, LPL, FAS, GLUT4, and ACO increased up to thousands of folds during 3T3-L1 differentiation compared to those untreated cells. Finally, the reporter gene assay confirmed that CE increased transactivities of both full length and LBD of PPAR*γ* and PPAR*α* significantly.

Diet-induced obese mouse is an early type 2 diabetic model. After 5 months induction with 60% calories fat diet, the body weight and fat weight of C57BL/6J mice were increased remarkably, and the mice developed insulin resistance and hyperinsulinemia fatty liver, and impairment of liver function which are often observed in early type 2 diabetes [17, 18]. Our results showed that after 3 weeks gavage with CE, both IPGTT and hyperinsulinemia improved markedly compared to the untreated ones. Although both increased insulin secretion and reduced insulin resistance can improve IPGTT, lowered insulin level and improved IPGTT in our study are beneficial to the reduction of insulin resistance. Serum contents of FFA, which impair insulin secretion and induce beta-cell lipotoxicity [19] and inhibit insulin-stimulated glucose uptake into muscle [20], were also reduced notably in DIO mice. The gene expression of PPAR*γ* and its target genes CD36, LDL in white fat tissue, and PPAR*α* and its target gene ACO in liver were also elevated in CE-treated DIO mice indicating that CE may act as a dual activator of PPAR*γ* and PPAR*α* resulting in improved insulin resistance and lowered serum lipids. In genetic diabetic model *db*/*db* mice, CE played similar roles in hypoglycemia and hypolipidemia.

Type 2 diabetic patients are at a higher risk for cardiovascular disease (CVD) than nondiabetic individuals [21]. Current evidence overwhelmingly confirms the role of low-density lipoprotein cholesterol (LDL-c) in the pathogenesis of atherosclerosis and the risk of CVD, lowering LDL-c is associated with a reduction in risk of CVD [22, 23]. Our study shows that CE reduces serum LDL-c to normal level in DIO mice, suggesting that CE may be beneficial to CVD.

Another finding may be noted in this study is that CE treatment improved impaired liver function in DIO mice. Elevated serum levels of AST and the ratio of AST/ALT usually indicate hepatocyte damage [24], and the most common presentation is elevated liver enzymes AST and ALT in fatty liver [25]. In DIO mice, serum AST and ALT were increased notably, but 3 weeks of CE treatment significantly decreased serum AST level and the ratio of AST/ALT, suggesting that CE may play an important role in improving liver function.

A meta-analysis based on five randomized clinical trials have shown that the use of cinnamon does not appear to improve A1C, FBG, or lipid parameters in patients with type 1 or type 2 diabetes [26]. Our preliminary study also showed no effect on DIO or *db*/*db* mice when cinnamon powder was used without water extraction. In present study, cinnamon water extract exhibited hypoglycemic and hypolipidemic effects on both DIO and *db*/*db* mice. This is in agreement with the results reported by Mang et al. demonstrating that the aqueous cinnamon extract seemed to have a moderate effect in reducing fasting plasma glucose concentrations in diabetic patients with poor glycaemic control [27]. The water extraction may have enriched the active components of cinnamon to activate both PPAR*α* and PPAR*γ* which are beneficial to lipid and glucose metabolism. Thus, preparation of cinnamon such as water extraction may be considered when designing new clinical trials.

In conclusion, our study demonstrated that CE had several beneficial effects on type 2 diabetes possibly through the activation of both PPAR*γ* and PPAR*α* resulting in improved insulin resistance, lowered blood glucose, and serum lipid level without weight gain and the structure change of the white adipose tissue. In addition, CE improved the liver function of obese mice. CE may have potential use in management of obesity-related type 2 diabetes and hyperlipidemia.

## Figures and Tables

**Table 1:** Effects of CE on serum chemistry of DIO mice. NFC: normal-fat diet control; HFC: high-fat diet control. The data are represented as mean ± SE, *n* = 5 for each group. *P < .05, **P < .01 compared to HFC.

Serum chemistry	NFC (H_2_O)	HFC (H_2_O)	HF + CE (400 mg/kg)
FFA (mmol/L)	1.31 ± 0.06	1.67 ± 0.15	1.08 ± 0.08**
TC (mmol/L)	1.46 ± 0.15	2.39 ± 0.33	2.22 ± 0.31
TG (mmol/L)	0.13 ± 0.08	0.14 ± 0.03	0.11 ± 0.07
HDL (mmol/L)	1.14 ± 0.14	2.04 ± 0.27	2.04 ± 0.24
LDL (mmol/L)	0.20 ± 0.03	0.26 ± 0.07	0.16 ± 0.08*
Insulin (ng/mL)	0.49 ± 0.07	1.44 ± 0.75	1.09 ± 0.25

AST (IU/L)	164.6 ± 83.7	330.8 ± 152.4	128.3 ± 27.4**

ALT (IU/L)	23.28 ± 10.8	86.43 ± 49.5	60.31 ± 15.99

## ABBREVIATIONS

ACO: Acyl-CoA oxidase
FAS: Fatty acid synthase
Glut4: Glucose transporting protein 4
LPL: Lipoprotein lipase
CD36: Fatty acid transporter
DIO: Diet-induced obesity
FFA: Free fatty acids
IPGTT: Intraperitoneal glucose tolerance test
HDL-C: High-density lipoprotein cholesterol
LDL-C: Low-density lipoprotein cholesterol
PPAR: Peroxisome proliferator-activated receptor
TC: Total cholesterol
TG: Triglyceride
TZD: Thiazolidinediones
GLU: Glucose
AST: Aspartate aminotransferase
ALT: Alanine aminotransferase
CE: Cinnamon water extract
LBD: Ligand-binding domain
DM: Differentiated medium.

## References

[1] DeFronzo RA, Ferrannini E (1991). Insulin resistance: a multifaceted syndrome responsible for NIDDM, obesity, hypertension, dyslipidemia, and atherosclerotic cardiovascular disease. *Diabetes Care*.

[2] Desvergne B, Wahli W (1999). Peroxisome proliferator-activated receptors: nuclear control of metabolism. *Endocrine Reviews*.

[3] Steiner G, Hamsten A, Hosking J (2001). Effect of fenofibrate on progression of coronary-artery disease in type 2 diabetes: the Diabetes Atherosclerosis Intervention Study, a randomised study. *The Lancet*.

[4] Olefsky JM (2000). Treatment of insulin resistance with peroxisome proliferator-activated receptor *γ* agonists. *The Journal of Clinical Investigation*.

[5] Cleeman JI (2001). Executive summary of the third report of the National Cholesterol Education Program (NCEP) expert panel on detection, evaluation, and treatment of high blood cholesterol in adults (adult treatment panel III). *The Journal of the American Medical Association*.

[6] Kumar S, Boulton AJM, Beck-Nielsen H (1996). Troglitazone, an insulin action enhancer, improves metabolic control in NIDDM patients. *Diabetologia*.

[7] Mayerson AB, Hundal RS, Dufour S (2002). The effects of rosiglitazone on insulin sensitivity, lipolysis, and hepatic and skeletal muscle triglyceride content in patients with type 2 diabetes. *Diabetes*.

[8] Miyazaki Y, Mahankali A, Matsuda M (2001). Improved glycemic control and enhanced insulin sensitivity in type 2 diabetic subjects treated with pioglitazone. *Diabetes Care*.

[9] Chitturi S, George J (2002). Hepatotoxicity of commonly used drugs: nonsteroidal anti-inflammatory drugs, antihypertensives, antidiabetic agents, anticonvulsants, lipid-lowering agents, psychotropic drugs. *Seminars in Liver Disease*.

[10] Rendell MS, Kirchain WR (2000). Pharmacotherapy of type 2 diabetes mellitus. *The Annals of Pharmacotherapy*.

[11] Khan A, Safdar M, Khan MMA, Khattak KN, Anderson RA (2003). Cinnamon improves glucose and lipids of people with type 2 diabetes. *Diabetes Care*.

[12] Kim SH, Hyun SH, Choung SY (2006). Anti-diabetic effect of cinnamon extract on blood glucose in db/db mice. *Journal of Ethnopharmacology*.

[13] Talpur N, Echard B, Ingram C, Bagchi D, Preuss HG (2005). Effects of a novel formulation of essential oils on glucose-insulin metabolism in diabetic and hypertensive rats: a pilot study. *Diabetes, Obesity and Metabolism*.

[14] Roffey B, Atwal A, Kubow S (2006). Cinnamon water extracts increase glucose uptake but inhibit adiponectin secretion in 3T3-L1 adipose cells. *Molecular Nutrition & Food Research*.

[15] Anderson RA, Broadhurst CL, Polansky MM (2004). Isolation and characterization of polyphenol type-A polymers from cinnamon with insulin-like biological activity. *Journal of Agricultural and Food Chemistry*.

[16] Huang C, Zhang Y, Gong Z (2006). Berberine inhibits 3T3-L1 adipocyte differentiation through the PPAR*γ* pathway. *Biochemical and Biophysical Research Communications*.

[17] Ahrén B, Simonsson E, Scheurink AJW, Mulder H, Myrsén U, Sundler F (1997). Dissociated insulinotropic sensitivity to glucose and carbachol in high-fat diet—induced insulin resistance in C57BL/6J mice. *Metabolism*.

[18] Winzell MS, Ahrén B (2004). The high-fat diet-fed mouse: a model for studying mechanisms and treatment of impaired glucose tolerance and type 2 diabetes. *Diabetes*.

[19] Kashyap S, Belfort R, Gastaldelli A (2003). A sustained increase in plasma free fatty acids impairs insulin secretion in nondiabetic subjects genetically predisposed to develop type 2 diabetes. *Diabetes*.

[20] Boden G (1999). Free fatty acids, insulin resistance, and type 2 diabetes mellitus. *Proceedings of the Association of American Physicians*.

[21] Betteridge J (2005). Benefits of lipid-lowering therapy in patients with type 2 diabetes mellitus. *The American Journal of Medicine*.

[22] Collins R, Armitage J, Parish S, Sleight P, Peto R (2002). MRC/BHF Heart Protection Study of cholesterol lowering with simvastatin in 20 536 high-risk individuals: a randomised placebo-controlled trial. *The Lancet*.

[23] Downs JR, Clearfield M, Weis S (1998). Primary prevention of acute coronary events with lovastatin in men and women with average cholesterol levels: results of AFCAPS/TexCAPS. *The Journal of the American Medical Association*.

[24] Renner EL, Dällenbach A (1992). Increased liver enzymes: what should be done?. *Therapeutische Umschau*.

[25] O'Connor BJ, Kathamna B, Tavill AS (1997). Nonalcoholic fatty liver (NASH syndrome). *The Gastroenterologist*.

[26] Baker WL, Gutierrez-Williams G, White CM, Kluger J, Coleman CI (2008). Effect of cinnamon on glucose control and lipid parameters. *Diabetes Care*.

[27] Mang B, Wolters M, Schmitt B (2006). Effects of a cinnamon extract on plasma glucose, HbA_1c_, and serum lipids in diabetes mellitus type 2. *European Journal of Clinical Investigation*.

